# Transcriptional Profiling of *Bacillus anthracis* Sterne (34F_2_) during Iron Starvation

**DOI:** 10.1371/journal.pone.0006988

**Published:** 2009-09-21

**Authors:** Paul E. Carlson, Katherine A. Carr, Brian K. Janes, Erica C. Anderson, Philip C. Hanna

**Affiliations:** 1 Department of Microbiology and Immunology, University of Michigan Medical School, Ann Arbor, Michigan, United States of America; Charité-Universitätsmedizin Berlin, Germany

## Abstract

Lack of available iron is one of many environmental challenges that a bacterium encounters during infection and adaptation to iron starvation is important for the pathogen to efficiently replicate within the host. Here we define the transcriptional response of *B. anthracis* Sterne (34F_2_) to iron depleted conditions. Genome-wide transcript analysis showed that *B. anthracis* undergoes considerable changes in gene expression during growth in iron-depleted media, including the regulation of known and candidate virulence factors. Two genes encoding putative internalin proteins were chosen for further study. Deletion of either gene (GBAA0552 or GBAA1340) resulted in attenuation in a murine model of infection. This attenuation was amplified in a double mutant strain. These data define the transcriptional changes induced during growth in low iron conditions and illustrate the potential of this dataset in the identification of putative virulence determinants for future study.

## Introduction

*Bacillus anthracis*, the causative agent of anthrax, is a highly virulent pathogen that has been used in recent history as a biological weapon [1]. In the environment, *B. anthracis* exists primarily as an infectious spore, which can remain dormant for many years [2]. Upon infection of a mammalian host, the spores are believed to associate with regional phagocytes, rapidly germinate and begin to divide [3], [4]. Following these early events, vegetative *B. anthracis* escape the phagocytes and replicate in the lymphatic system prior to entering the bloodstream [5]. Titers can reach as high as 10^7^ or 10^8^ bacteria per milliliter of blood. This high bacterial load can lead to septicemia, toxemia, and eventually the death of the host [5]. The success of *B. anthracis* as a pathogen is at least partly due to the development of mechanisms allowing the rapid transition from environmental dormancy to active replication within the host. A better understanding of the mechanisms involved in this adaptation could aid in the development of improved treatment options and vaccines.

Pathogens encounter a variety of signals during infection of a mammalian host. Our laboratory has previously shown that the macrophage environment in which *B. anthracis* exists during early infection induces significant transcriptional changes in the bacterium, presumably altering the protein profiles of the organism for optimal growth in this environment [6]. Earlier studies have also examined the response of this pathogen to oxidative stress, a specific signal that the bacterium is likely to encounter during its time within the macrophage cytosol [7]. Also, another host-related signal, increased carbon dioxide levels is known to alter expression of the *B. anthracis* toxin genes as well as others [8], [9], [10].

Though there is a substantial body of work examining the response of *B. anthracis* to several signals encountered in the host, the response of the pathogen to iron starvation remains unstudied on a global scale. In the human host, the concentration of free iron available to the bacterium is limiting for growth [11]. Effective strategies for adaptation to this altered environmental condition and, subsequently, the acquisition of iron, are vital to the survival of most bacterial pathogens. Many pathogens undergo significant changes in their gene and protein expression to adapt to growth in iron limiting conditions, including *Francisella tularensis*, *Mycobacterium tuberculosis*, and *Streptococcus pneumoniae* [12], [13], [14].

Here we report the transcriptional response of *B. anthracis* grown in iron limiting conditions. When compared to bacteria that were grown in iron replete conditions, significant changes in gene expression were observed as early as 2 hours post-inoculation. Clear patterns of gene regulation were observed with significant changes in genes involved in a wide range of processes being seen. One set of differentially regulated genes, annotated in genome sequence databases as internalins, were chosen for further study. These data fill a significant gap in our current knowledge of *B. anthracis* pathogenesis and provide the basis for examination of specific mechanisms of iron acquisition and general pathogenesis of this organism.

## Results and Discussion

### Response of *B. anthracis* Sterne to iron starvation

**Figure 1:**
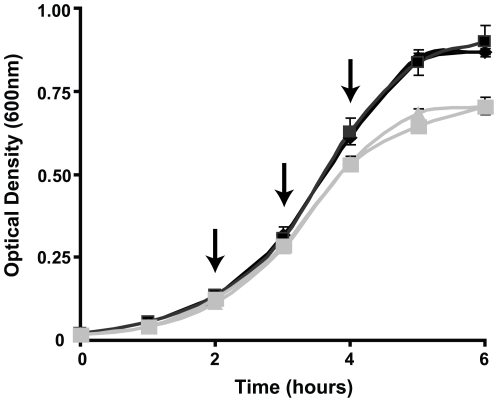
Growth of *B. anthracis* in iron depleted media. *B. anthracis* vegetative cells were used to inoculate either IRM (black lines) or IDM (gray lines) at an initial OD_600_ = 0.05. Growth was monitored over time by measuring change in OD_600_. RNA was harvested at two, three, and four hours after inoculation (arrows).

In the study reported here, we sought to define the response of *B. anthracis* to low iron concentrations, a signal that mimics conditions encountered by the bacterium within a mammalian host. Growth characteristics of *B. anthracis* in iron depleted media (IDM) has been previously reported [15], [16]. We first wanted to examine the growth kinetics of this pathogen in media containing high and low concentrations of iron to identify optimal timepoints for RNA isolation. Vegetative bacilli were inoculated into either IDM or iron replete media (IRM) at an initial OD_600_ of 0.05 and growth was monitored hourly (Figure 1). Based on the growth characteristics of the bacteria in these media, hourly intervals from 2 to 4 hours were chosen as time points for RNA isolations (Figure 1–arrows). The growth rate of *B. anthracis* in IDM slowed noticeably beyond the four hour timepoint, with entrance into stationary phase apparent by five hours. Because of the growth phase difference between the two conditions (late log for IRM and early stationary for IDM) the five hour timepoint was not included in the microarray comparison.

In order to gain a thorough understanding of the response of *B. anthracis* to iron starvation, we utilized DNA microarrays to examine differences in transcriptional profiles following growth in either IDM or IRM. RNA was isolated from *B. anthracis* following 2, 3, or 4 hours of growth in either IDM or IRM. Each of the RNA samples (four replicates were performed for each timepoint) was then subjected to microarray analysis using a custom *B. anthracis* Affymetrix GeneChip [6], [7], [17]. Statistically significant differences in gene expression between *B. anthracis* grown in high (IRM) or low (IDM) iron concentrations were defined as a J5 score greater than two as well as a fold change greater than two (IDM vs. IRM). The J5 statistical test was chosen for its stringency when looking at a data set with a low *n*, limiting the chance of picking up false positives [18]. Statistical comparisons were performed at each of the three timepoints independently. At two hours the differences observed were minimal, with only 41 genes exhibiting statistically significant changes based on the above criteria. Beyond two hours, more significant changes were observed, presumably as the trace amounts of iron remaining in the IDM were consumed by the bacteria. At the three and four hour timepoints, significant changes were observed in the expression levels of 124 and 282 genes, respectively. The majority of the genes that exhibited significant changes in expression level at two hours were also observed to be significant at both three and four hours (87.8%). In addition, 86.3% of the genes identified as significant at three hours were significantly altered at the four hour timepoint as well.

**Figure 2:**
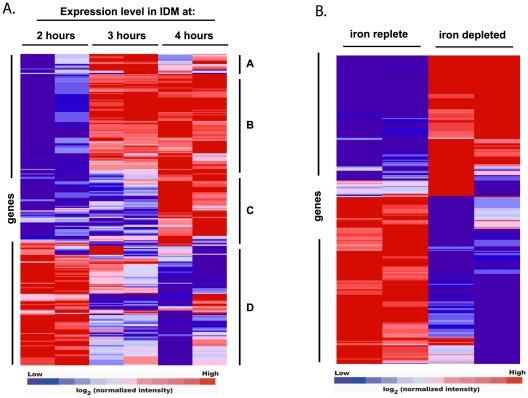
Changes in *B. anthracis* gene expression induced by iron starvation over time. Hierarchical clustering of genes identified as statistically significant by J5 test across two independent microarray experiments. Fluorescence intensities were normalized as described in the methods. Normalized intensity values from two experiments were input into Gene Pattern together and clustered using the Pearson correlation. Genes clustered into four groups, induced with a peak at three hours (A), induced at three and four hours (B), induced at four hours (C), and repressed (D). Data shown are normalized intensity values over time for samples grown in IDM only.

Those genes exhibiting significantly altered expression following four hours of growth in IDM were put into Gene Pattern [19] for hierarchical clustering (Figure 2). Figure 2A shows a clustering of log_2_ transformed normalized intensity values of all gene found to be significantly changed at four hours. Data for the entire timecourse in IDM are shown for these genes to illustrate the observed changes over time in this medium. The clustered data demonstrate a clear pattern of transcriptional regulation over time. Cluster A represents a small subset of genes that appear to peak at three hours, although their expression remains significantly higher at four hours (compared to IRM). Cluster B represent genes that peak at three hours and remain highly expressed through the end of the experiment while cluster C represents genes with a peak in expression at four hours. Finally, cluster D consists of those genes exhibiting a decrease in expression during iron starvation.

We next sought to compare the gene expression profiles between IDM and IRM at this single timepoint (Figure 2B). Here the data cluster into two distinct groups, with 136 genes up-regulated and 145 genes down-regulated. Since the majority of the genes differentially regulated at earlier timepoints were also identified at four hours, all subsequent analyses will focus on the genes identified at the final timepoint. The top 50 genes that were up-regulated after four hours in IDM are listed in **Table 1**. The complete list of significant genes identified at four hours, along with those for the other two timepoints, can be found in the supplementary information (Table S1–up-regulated genes Table S2 - down-regulated genes).

**Table 1:** Fifty genes most induced during iron starvation.

Gene	J5	FC	annotation
GBAA4788	23.8	160.6	hypothetical protein GBAA4788
GBAA4789	22.4	120.7	cell wall anchor domain-containing protein
GBAA4786	22.4	120.1	iron compound ABC transporter, iron compound-binding protein
GBAA4787	22.1	112.1	hypothetical protein GBAA4787
GBAA2368	20.5	79.7	2,3-dihydroxybenzoate-2,3-dehydrogenase
GBAA1393	20.2	74.2	hypothetical protein GBAA1393
GBAA2369	19.6	65.4	isochorismate synthase DhbC
GBAA4785	19.2	60.7	iron compound ABC transporter, permease protein
GBAA4783	19.2	60.4	hypothetical protein GBAA4783
GBAA2370	18.6	53.3	2,3-dihydroxybenzoate-AMP ligase
GBAA1345	18.5	52.0	pseudogene - conserved hypothetical protein
GBAA1394	18.4	50.4	flavodoxin
GBAA4784	18.2	48.5	iron compound ABC transporter, ATP-binding protein
GBAA2374	18.1	48.2	EmrB/QacA family drug resistance transporter
GBAA2371	18.0	46.7	isochorismatase
GBAA1395	17.8	44.9	hypothetical protein GBAA1395
GBAA2376	17.7	44.0	hypothetical protein GBAA2376
GBAA2373	17.4	41.3	mbtH-like protein
GBAA2372	16.7	35.5	nonribosomal peptide synthetase DhbF
GBAA2375	16.2	32.1	4′-phosphopantetheinyl transferase, putative
GBAA4597	16.1	30.9	iron compound ABC transporter, iron compound-binding protein
GBAA1346	15.9	29.9	internalin, putative
GBAA4782	14.3	21.2	heme-degrading monooxygenase IsdG
GBAA4596	13.9	19.4	iron compound ABC transporter, permease protein
GBAA1093	13.7	18.8	S-layer protein, putative
GBAA4595	12.8	15.5	iron compound ABC transporter, ATP-binding protein
GBAA0966	12.4	14.1	hypothetical protein GBAA0966
GBAA4594	12.3	13.8	ankyrin repeat-containing protein
GBAA2255	12.1	13.2	substrate-binding family protein, putative
GBAA0552	12.0	13.0	internalin, putative
GBAA3595	12.0	12.9	BNR repeat-containing protein
GBAA3596	11.0	10.6	flavodoxin
GBAA3231	10.3	9.1	hypothetical protein GBAA3231
GBAA4781	10.0	8.5	sodium/hydrogen exchanger family protein
GBAA5690	9.5	7.6	murein hydrolase regulator LrgA
GBAA_pXO1_0119	9.1	7.0	hypothetical protein
GBAA3534	8.9	6.8	iron compound ABC transporter, permease protein
GBAA5689	8.7	6.4	antiholin-like protein LrgB
GBAA3533	8.7	6.4	iron compound ABC transporter, permease protein
GBAA2367	8.5	6.1	oxalate:formate antiporter, putative
GBAA3531	8.5	6.1	iron compound ABC transporter, iron compound-binding protein, putative
GBAA_pXO1_0120	8.4	6.1	hypothetical protein
GBAA1092	8.2	5.7	hypothetical protein GBAA1092
GBAA0766	8.1	5.7	nitroreductase family protein
GBAA3866	7.8	5.3	iron compound ABC transporter, permease protein
GBAA3532	7.6	5.1	hypothetical protein GBAA3532
GBAA3867	7.5	4.9	pseudogene - iron compound ABC transporter, iron compound-binding protein
GBAA3865	7.4	4.9	iron compound ABC transporter, permease protein
GBAA2688	7.3	4.8	hypothetical protein GBAA2688
GBAA3863	7.3	4.8	hypothetical protein GBAA3863

### Validation of microarray results using quantitative real time PCR

**Figure 3:**
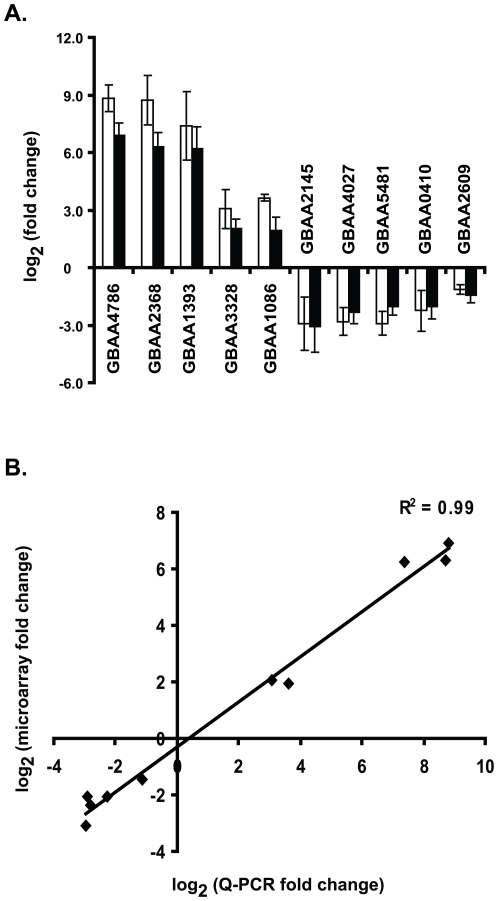
Validation of microarray data. (A). Gene expression changes measured by Q-PCR at four hours. RNA from four experiments was tested for specific genes using real time PCR. The results of Q-PCR are represented with white bars, while corresponding values from the microarray experiments are represented with black bars. Data are presented as mean±SEM of log_2_ transformed fold change where fold change is the ratio of expression in IDM to IRM. (B). Correlation analysis comparing fold change between microarray and Q-PCR for ten *B. anthracis* genes. Data are plotted as log_2_ transformed microarray data compared to log_2_ transformed Q-PCR data. The correlation coefficient (R^2^) between these two analyses was 0.99.

In order to confirm the microarray results, ten genes were chosen (five up-regulated and five down-regulated) for analysis using quantitative real time PCR (Q-PCR). Q-PCR experiments were carried out on RNA isolated following four hours of growth in IDM or IRM. Among the up-regulated genes tested were GBAA4786, GBAA2368, GBAA1393, GBAA3328, and GBAA1086. Down-regulated genes used to confirm the microarray analysis included GBAA2145, GBAA4027, GBAA5481, GBAA0410, and GBAA2609. When measured by Q-PCR, each of these genes exhibited a pattern of expression nearly equal to what was observed on the microarrays (Figure 3A white bars = microarray data; black bars = Q-PCR). A correlation plot comparing microarray data to those generated using Q-PCR demonstrated a strong positive association between these two data sets (Figure 2B). Taken together, these data validate the microarray results, solidifying the findings reported above.

### Analysis of genes based on COG classification

**Figure 4:**
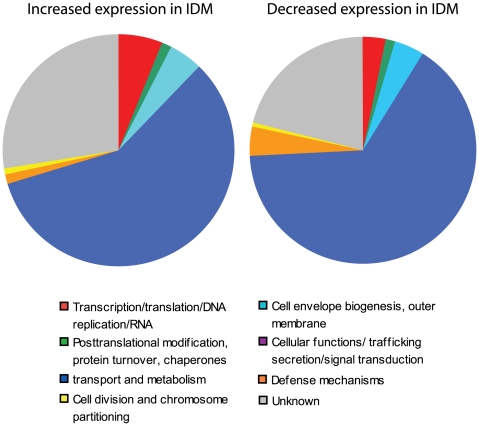
Categorical analysis of genes induced during iron starvation. Pie charts represent the COG classification for the genes uniquely regulated in the conditions listed. Each pie represents 100% of the genes exhibiting significant gene expression changes following growth in iron depleted media. COG categories were identified for each differentially regulated gene based on genomic annotation (GenBank accession, NC_007530). To simplify the analysis, the COG categories were grouped into general categories as follows: Categories A, J, K, L, and RNA genes were merged into “Transcription/translation/DNA replication/RNA”; C, E, F, G, H, I, P, and Q were combined into “Transport and metabolism”; COGs N, T, and U were combined into “Cellular functions/trafficking/secretion/signal transduction”; and categories R and S were grouped with uncategorized genes and classified as “Unknown.” Other COG categories listed are reported as they were originally defined.

In order to more thoroughly examine these results, the uniquely regulated genes were grouped based on the annotated COGs (Clusters of Orthologous Groups). Related COGs were grouped together into the categories shown in Figure 4. The pie charts show the categorical designation of the unique genes that are induced or repressed after four hours in IDM (Figure 4). Each chart represents 100% of the COG designations for the genes that were induced or repressed as discussed above. Each slice within a given chart represents the percentage of genes identified from a given category. By examining these general relationships between genes regulated by iron starvation, many interesting observations can be made.

**Figure 5:**
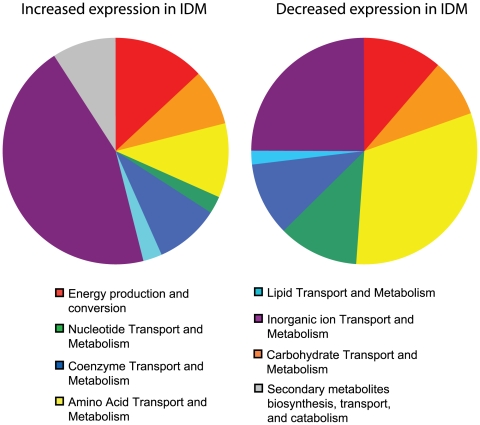
Specific analysis of metabolic changes induced in *B. anthracis* during iron starvation. Each pie represents 100% of the metabolism related genes exhibiting significant gene expression changes following growth in iron depleted media (blue slice from Figure 4). Specific COG categories were identified for each gene based on genomic annotation (accession, NC_007530) and are listed as they were originally defined.

Immediately, it was clear that the most striking changes, up- and down-regulated, were in genes involved in transport and metabolism, with this category comprising nearly 75% of each pie (Figure 4, dark blue slice). Because each of these slices was identical, it was difficult to determine what changes were occurring in this group. To examine this more closely, we looked at each of the COGs that was used to make up the ‘transport and metabolism’ group in Figure 4. Figure 5 shows the pie charts with this more specific categorical representation of the COG designations for the metabolic genes either induced (76) or repressed (96) in IDM. Here we can see clear differences in the genes involved in these transport and metabolic pathways following growth under iron depeleted conditions. The COG ‘inorganic ion transport and metabolism’ (purple slice) represents 42% of the total COG designations for genes upregulated in IDM. The genes up-regulated in this category encode orthologs of proteins with annotated roles in iron acquisition, while those being repressed during iron starvation are genes involved in transport of other ions including phosphate and nitrate. In fact, among the genes most up-regulated in IDM was the *B. anthracis isd* locus (GBAA4782–4789), which is known to be regulated by environmental iron concentration through the presence of known ferric regulatory regions (FUR boxes) [20]. This locus encodes proteins involved in iron binding, uptake, and release [20]. Work with the virulent Vollum strain of *B. anthracis* has shown that three genes from this locus (GBAA4787–4789) are not required for virulence in the guinea pig model of infection [21]. However, the fact that other members of this operon have been shown to play a role in iron acquisition from heme, indicates that some details about the role of the *isd* locus in *B. anthracis* virulence may remain to be elucidated [22].

Components of another other iron uptake system of specific interest, encoded by GBAA5328–5330, were induced by iron starvation. It has recently been shown that a *B. cereus* homolog of the protein encoded by GBAA5330 is able to bind to the siderophore petrobactin [23]. Similar binding properties were observed for another iron binding protein, GBAA4766, the expression of which was also up-regulated during growth in IDM [23]. Synthesis of petrobactin is vital to *B. anthracis* pathogenesis and mutants unable to produce this molecule are significantly impaired for growth in IDM and attenuated in a mouse model of infection [15], [24]. This is in sharp contrast to mutants unable to synthesize bacillibactin, the other siderophore produced by *B. anthracis*, which grows well in IDM and shows no significant attenuation in a mouse model of infection [15]. Unexpectedly, the genes that comprise the petrobactin biosynthesis pathway were not significantly up-regulated during growth in IDM. In light of these data, it is not surprising that expression of the uptake systems for this siderophore would be induced, potentially enabling the bacterium to acquire more iron-containing petrobactin. In fact, we have shown that one of these genes is important in the pathogenesis of *B. anthracis* and is required for growth in IDM, as well as for full murine virulence (Carlson et. al. manuscript submitted).

Another group of metabolic genes that were induced during iron starvation were the flavodoxins (GBAA1394 and GBAA3596). These genes, which are part of the ‘energy production and conversion’ category (Figure 5, red slices), appear to be co-regulated with adjacent hypothetical proteins. Flavodoxins are involved in a variety of reactions in bacteria and are essential in many bacterial species [25]. These proteins have previously been shown to replace ferrodoxins in NADP^+^ and N_2_ reduction reactions during growth in low iron conditions [25]. Not surprisingly, our microarray data show that *B. anthracis* increases the expression of flavodoxins and decreases expression of ferrodoxins during growth in iron depleted conditions (Tables S1 and S2). Interestingly, flavodoxins have previously been associated with *B. anthracis* infection, being up-regulated following growth in macrophages [6]. Our data, along with those previously published, provide increasing evidence of a role for flavodoxins during *B. anthracis* infection of mammalian hosts. The specific role of these proteins is currently under investigation.

Another category of specific interest, which may be related to the metabolic changes observed, includes genes involved in the control of transcription and translation (Figure 4, red slices). Altered regulation of a single transcription factor in response to an environmental signal could lead to significant changes in the transcriptome of the organism. Although only few members of this category exhibit altered regulation during iron starvation (8 up-regulated genes and 5 down-regulated), the downstream effects of these proteins could be significant. Among the transcription factors differentially regulated during iron starvation are members of the AraC, LacI, and GntR families of transcriptional regulators. Proteins from each of these families play a role in carbon catabolite repression, a process known to be regulated by the presence or absence of environmental carbon sources [26], [27], [28]. Although the presence of genes thought to play a role in CCR is interesting, the regulation of these genes in this particular system (iron starvation) seems unusual. For the experiments presented here, the carbon composition of the two media types compared here, IDM and IRM, is identical. The differential regulation of these genes may indicate either a novel mechanism of metabolic control or, more likely, a function for these particular transcription factors unrelated to CCR.

Taken together, the data on uptake systems, transcription factors, and general metabolism indicate a significant change in the global metabolism of *B. anthracis* growing under iron limiting conditions. Further evaluation of the metabolic and transport pathways induced in *B. anthracis* during iron starvation, and other host specific cues, may provide insight into the specific metabolic changes the bacterium requires for adaptation to life within a mammalian host. Further characterization of these changes may lead to the development of treatments that target specific aspects of *B. anthracis* metabolism within the host, thereby preventing the organism from thriving in the host environment.

A significant portion of the genes differentially regulated in iron depleted conditions do not have a known function (Figure 4, gray slice). Approximately 40% of the *B. anthracis* Sterne genome does not have an annotated function, so it is expected that we would see a significant number of these genes being differentially regulated under most conditions. Searching through this list, however, should provide some interesting candidates for future study. Many of the hypothetical proteins encoded by the *B. anthracis* genome are conserved across bacterial species; however there are genes which are specific to members of the *Bacillus* genus. Many genes induced by low iron levels, including GBAA1395, GBAA1458, and GBAA2286 encode proteins that do not share significant homology with proteins outside of the pathogenic *Bacillus* species. Genes such as GBAA1092 and the plasmid encoded GBAA_pXO1_0120 are of particular interest as they have no significant homology outside of *B. anthracis*. Further evaluation of these genes, specifically those up-regulated in conditions that mimic a host environment (*e.g.* iron starvation), may facilitate the identification of novel findings regarding mechanisms of *B. anthracis* pathogenesis.

### Attenuation of internalin deficient *B. anthracis*

Two other genes of unknown function that were induced during iron starvation, GBAA0552 and GBAA1346. These genes have been annotated as putative internalins based on the presence of leucine rich repeat (LRR) domains within the predicted protein sequence [29]. These LRR domains are found in all *Listeria* internalins and are known to facilitate the interaction with their respective host-cell receptors [29]. The protein encoded by GBAA0552 also contains a NEAT domain, which has been associated with heme binding properties in other *B. anthracis* proteins [22]. The protein encoded by GBAA1346 lacks this domain, but contains three SLH domains, commonly found in protein that are anchored to the S-layer of the cell wall [30]. Among *Bacillus* species, these genes are only found within the pathogenic members, though their potential role in virulence remains to be elucidated [31]. To examine the potential role of these proteins in *B. anthracis* growth during iron starvation, we created non-polar deletion mutants lacking either one or both of these putative internalins. Curiously, none of these mutants exhibited a reduced ability to grow in IDM (data not shown).

Homologs of these putative internalin genes were identified as candidate *B. cereus* virulence factors due to their expression during infection of insects [32]. Although the function of these proteins in *B. anthracis* is currently unknown, internalins play an important role in the virulence of other pathogens, though their mechanisms of action remain unclear [29], [31], [33], [34]. In *Listeria monocytogenes*, some internalins are known to promote invasion into epithelial cells through the binding of E-cadherin expressed on the host cell surface [33], [34]. Interestingly, increased expression of these genes in *B. anthracis* also was observed during oxidative stress, which is another condition the bacterium would encounter during infection of a mammalian host [7].

**Figure 6:**
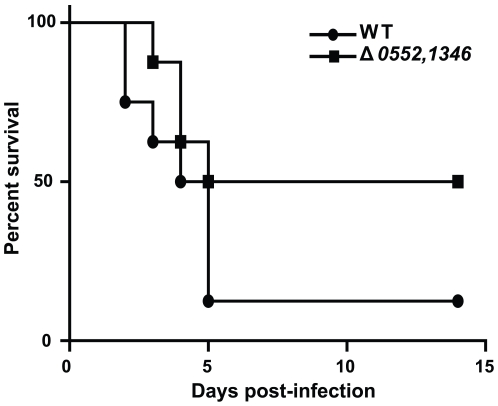
Attenuation of *B. anthracis* Δ0552, 1346 strain lacking both putative internalins. DBA/2J mice were infected by intratracheal infection with WT (circles) or Δ0552, 1346 (squares) spores at 1.5×10^5^ spores per mouse. Mice were monitored for fourteen days, then all surviving mice were euthanized.

Although no differences in IDM growth were observed for these mutants, the combination of *B. anthracis* gene expression data and the fact that internalin-like proteins have been clearly linked to virulence in other pathogens led us to examine the role of these two genes in *B. anthracis* virulence. We sought to determine if *B. anthracis* strains lacking these putative internalins were attenuated for virulence in a mouse model of inhalational anthrax. Wild-type *B. anthracis*, Δ0552, Δ1346, and Δ0552/1346 strains were inoculated intratracheally into DBA/2J mice at various doses to determine the LD_50_ of each strain. The single deletion strains both exhibited attenuation in this model, with the loss of either GBAA0552 or GBAA1346 resulting in an increase in LD_50_ of approximately 110 and 60 times that of wild-type spores, respectively (Table 2). The double mutant exhibited the highest degree of attenuation, with an LD_50_ 160 times that of wild-type *B. anthracis*. A representative survival curve for the double mutant is shown in Figure 6. This level of attenuation is comparable with what has been observed for internalin mutants in *L. monocytogenes* [35], [36], [37]. Further study will be required to fully elucidate the role of these proteins in the pathogenesis of *B. anthracis*.

**Table 2:** Attenuated virulence of internalin mutants in DBA/2J mice.

Strain	LD50
wild-type	7.5×10^3^
Δ*0552*	8.4×10^5^
Δ*1346*	4.7×10^5^
Δ0552, 1346	1.2×10^6^

Collectively, the data presented here show significant changes in the transcriptional profile of *B. anthracis* during growth in iron limiting conditions. Significant increases in iron transport mechanisms and genes involved in various aspects of metabolism indicate adaptation to growth in these conditions. These data fill a gap in our current knowledge about the pathogenesis of this organism and provide the basis for future work examining the role of these genes during *B. anthracis* infection, as well as the potential of these genes to be utilized as the target of novel therapeutics.

## Materials and Methods

### Bacterial cultivation

All of the work described in this manuscript was performed using the Sterne 34F_2_ (pXO1^+^, pXO2^−^) strain of *B. anthracis*. Initial spore stocks were prepared as follows. A single *B. anthracis* culture was grown overnight at 37°C, and then 1 ml was used to inoculate 500 ml of G medium as described previously [35]. For all iron depletion studies, iron depleted media (IDM) was used [15]. Growth curve and RNA isolation experiments were performed as follows. Spores were germinated in brain heart infusion (BHI) broth and incubated overnight at room temperature. The following day, vegetative bacilli were diluted 1∶100 in fresh BHI and grown 1–2 hours. Early log-phase bacilli were then washed 3x with PBS and 3x with IDM to ensure removal of nutrients and potential iron sources carried over from the BHI. Washed bacteria were then used to inoculate cultures of IDM or iron replete media (IRM) [IDM+20 µM ferrous sulfate] at an OD_600_ = 0.05.

### RNA isolation and analysis

RNA isolations were performed as previously described [17]. Briefly, at indicated timepoints, cultures were passed over a 0.2 µM filter. Bacteria were resuspended from the filter with ice cold nuclease free water. Lysis buffer (2% SDS, 10 mM EDTA, 200 mM NaCl) was immediately added to the resuspended bacteria and the mixture was placed in a boiling water bath for three minutes. Nucleic acid was subsequently isolated through two hot phenol extractions (65°C) followed by phenol:chloroform and chloroform extractions (22°C). The isolated nucleic acids were then precipitated overnight at −20°C following the addition of 0.1 vol. ammonium acetate and 2.5 vol. ethanol. Resulting RNA was treated with DNase according to manufacturer's protocol (Turbo DNA-*free*, Ambion). DNA-free RNA was then precipitated using ammonium acetate/ethanol. RNA quantity was measured by spectrophotometry and quality was confirmed at each step using an Agilent Bioanalyzer according to manufacturer's protocol.

### Microarray data analysis

Analysis of the microarray data was carried out using Gene Expression Data Analyzer (GEDA; http://bioinformatics.upmc.edu/GE2/GEDA.html) [18]. Briefly, this online software package was used for statistical comparisons of individual conditions at each timepoint. Data were grouped into categories, IDM or IRM, and subjected to the J5 statistical analysis, which is designed for data sets with limited numbers of replicates and reduces the chances of false positives [18]. Genes were considered statistically significant if they had a J5 score greater than 2 and exhibited a fold change greater than 2. Supplementary tables S1 and S2 report both the J5 value and fold change for genes that changed significantly at each timepoint. Genes meeting these requirements were then entered into GenePattern [19] and data are presented as a hierarchical clustering (Pearson correlation) of log_2_ transformed normalized intensity values for these genes. Our datasets have been deposited to the ArrayExpress database under the accession number E-MEXP-2272.

### Quantitative PCR

cDNA synthesis was performed using Superscript III (Invitrogen) and 1 µg of total RNA. Real time reactions were performed with a 1∶500 final dilution of template cDNA. Primer sets were designed for genes indicated using Primer 3 [36] and reactions were carried out on an BioRad ICycler real time machine using SYBR Green labeling according to manufacturer'r protocol (BioRad). The *acoB* gene (GBAA2775) was used as the internal reference, as it was observed to have no change in expression on microarray data (data not shown). The Q-PCR data are presented as log_2_ transformed fold change values (iron depleted/iron rich). Data are presented as the mean±SEM for 4 independent experiments.

### Creation of internalin mutants

Mutants were generated by allelic exchange, using the method of Janes and Stibiwitz [37]. Each mutant allele was designed to replace codons 10 through 14 with the following sequence: TAA TAG TGA GGA TCC. This introduced three in-frame stop codons followed by the recognition site for the restriction endonuclease BamHI (to facilitate screening of the mutant allele). All other codons of the gene remain wild-type, therefore the mutant is predicted to be non-polar. The double mutant was generated by the introduction of the GBAA1346 mutation into the Δ0552 mutant by the same method.

### Mouse infections

Intratracheal infections of DBA/2J mice (Jackson Laboratories) were performed as previously described [38]. Groups of eight mice were infected with either wild-type or mutant spores at a variety of doses ranging from 1.5×10^5^ through 1.5×10^8^ spores per mouse. Mice were monitored over a period of fourteen days. LD_50_ values were calculated using the Moving Average Interpolation program available at (http://falkow.stanford.edu/whatwedo/software/software.html) [39]. All mouse infections performed in compliance with the National Institutes of Health Guidelines on the Care and Use of Animals and an animal study protocol approved by the University of Michigan's Institutional Animal Care and Use Committee.

## Supporting Information

Table S1 Genes upregulated during iron starvation in wild-type B. anthracis. mRNA transcripts upregulated in IDM as compared to regulation in IRM
(0.35 MB DOC)

Table S2 Genes down-regulated during iron starvation. mRNA transcripts downregulated in IDM as compared to regulation in IRM
(0.24 MB DOC)
