# Supplementary material for: Transcriptional Profiling of Bacillus anthracis Sterne (34F2) during Iron Starvation
Source: PLoS One. 2009 Sep 21;4(9):e6988. doi: 10.1371/journal.pone.0006988 (PMC2742718; doi:10.1371/journal.pone.0006988)
Supplement: Table S1 — Genes upregulated during iron starvation in wild-type B. anthracis. mRNA transcripts upregulated in IDM as compared to regulation in IRM (0.35 MB DOC) [file pone.0006988.s001.doc]

| **Table S1: Genes upregulated during iron starvation in wild-type *B. anthracis*.** | | | |
| --- | --- | --- | --- |
|  |  |  |  |
| 2 hours: |  |  |  |
| **Gene** | **J5** | **FC** | **annotation** |
| GBAA2369 | 31.5 | 4.6 | isochorismate synthase DhbC |
| GBAA2370 | 30.9 | 4.5 | 2,3-dihydroxybenzoate-AMP ligase |
| GBAA2371 | 30.7 | 4.4 | isochorismatase |
| GBAA2368 | 30.0 | 4.3 | 2,3-dihydroxybenzoate-2,3-dehydrogenase |
| GBAA2374 | 28.7 | 4.0 | EmrB/QacA family drug resistance transporter |
| GBAA3595 | 27.3 | 3.7 | BNR repeat-containing protein |
| GBAA2373 | 26.5 | 3.6 | mbtH-like protein |
| GBAA1393 | 26.3 | 3.6 | hypothetical protein GBAA1393 |
| GBAA2372 | 26.2 | 3.6 | nonribosomal peptide synthetase DhbF |
| GBAA3596 | 26.1 | 3.5 | flavodoxin |
| GBAA1394 | 25.1 | 3.4 | flavodoxin |
| GBAA4789 | 24.8 | 3.3 | cell wall anchor domain-containing protein |
| GBAA4597 | 23.9 | 3.2 | iron compound ABC transporter, iron compound-binding protein |
| GBAA1395 | 23.3 | 3.1 | hypothetical protein GBAA1395 |
| GBAA3865 | 23.0 | 3.0 | iron compound ABC transporter, permease protein |
| GBAA4788 | 22.9 | 3.0 | hypothetical protein GBAA4788 |
| GBAA4596 | 22.8 | 3.0 | iron compound ABC transporter, permease protein |
| GBAA4595 | 22.6 | 3.0 | iron compound ABC transporter, ATP-binding protein |
| GBAA3866 | 22.6 | 3.0 | iron compound ABC transporter, permease protein |
| GBAA3863 | 22.5 | 3.0 | hypothetical protein GBAA3863 (Esterase_lipase domain) |
| GBAA2375 | 22.1 | 2.9 | 4'-phosphopantetheinyl transferase, putative |
| GBAA4594 | 22.0 | 2.9 | ankyrin repeat-containing protein |
| GBAA3864 | 21.9 | 2.9 | iron compound ABC transporter, ATP-binding protein |
| GBAA2376 | 21.0 | 2.8 | hypothetical protein GBAA2376 (Chorismate lyase domain) |
| GBAA3867 | 20.0 | 2.6 | pseudogene |
| GBAA4782 | 20.0 | 2.6 | heme-degrading monooxygenase IsdG |
| GBAA3533 | 18.2 | 2.4 | iron compound ABC transporter, permease protein |
| GBAA4786 | 18.1 | 2.4 | iron compound ABC transporter, iron compound-binding protein |
| GBAA3531 | 17.4 | 2.3 | iron compound ABC transporter, iron compound-binding protein, putative |
| GBAA1093 | 17.2 | 2.3 | S-layer protein, putative |
| GBAA3534 | 16.9 | 2.3 | iron compound ABC transporter, permease protein |
| GBAA3532 | 16.2 | 2.2 | hypothetical protein GBAA3532 |
| GBAA4652 | 15.9 | 2.2 | substrate-binding family protein, putative |
| GBAA0766 | 15.7 | 2.1 | nitroreductase family protein |
| GBAA1345 | 14.1 | 2.0 |  |
|  |  |  |  |
| 3 hours: |  |  |  |
| **Gene** | **J5** | **FC** | **annotation** |
| GBAA4788 | 38.8 | 260.3 | hypothetical protein GBAA4788 |
| GBAA4789 | 36.9 | 196.5 | cell wall anchor domain-containing protein |
| GBAA4786 | 33.7 | 125.8 | iron compound ABC transporter, iron compound-binding protein |
| GBAA4787 | 33.2 | 115.9 | hypothetical protein GBAA4787 |
| GBAA1393 | 32.8 | 109.4 | hypothetical protein GBAA1393 |
| GBAA2369 | 31.0 | 84.6 | isochorismate synthase DhbC |
| GBAA2368 | 30.9 | 84.2 | 2,3-dihydroxybenzoate-2,3-dehydrogenase |
| GBAA1394 | 30.3 | 77.0 | flavodoxin |
| GBAA1395 | 30.2 | 75.8 | hypothetical protein GBAA1395 |
| GBAA2370 | 29.9 | 72.4 | 2,3-dihydroxybenzoate-AMP ligase |
| GBAA2374 | 29.4 | 67.1 | EmrB/QacA family drug resistance transporter |
| GBAA2371 | 29.2 | 66.1 | isochorismatase |
| GBAA4783 | 29.1 | 64.6 | hypothetical protein GBAA4783 |
| GBAA4785 | 28.4 | 58.5 | iron compound ABC transporter, permease protein |
| GBAA2373 | 28.4 | 58.4 | mbtH-like protein |
| GBAA1345 | 28.2 | 56.6 | pseudogene |
| GBAA4784 | 27.6 | 52.1 | iron compound ABC transporter, ATP-binding protein |
| GBAA2372 | 26.9 | 47.0 | nonribosomal peptide synthetase DhbF |
| GBAA2376 | 25.7 | 39.5 | hypothetical protein GBAA2376 |
| GBAA4597 | 25.1 | 36.6 | iron compound ABC transporter, iron compound-binding protein |
| GBAA2375 | 24.0 | 31.1 | 4'-phosphopantetheinyl transferase, putative |
| GBAA1346 | 23.7 | 29.7 | internalin, putative |
| GBAA4596 | 23.3 | 28.1 | iron compound ABC transporter, permease protein |
| GBAA4782 | 23.3 | 28.0 | heme-degrading monooxygenase IsdG |
| GBAA4595 | 22.3 | 24.3 | iron compound ABC transporter, ATP-binding protein |
| GBAA4594 | 22.1 | 23.7 | ankyrin repeat-containing protein |
| GBAA1093 | 21.9 | 23.2 | S-layer protein, putative |
| GBAA3595 | 21.5 | 21.8 | BNR repeat-containing protein |
| GBAA3596 | 20.6 | 19.1 | flavodoxin |
| GBAA3865 | 16.7 | 10.9 | iron compound ABC transporter, permease protein |
| GBAA3863 | 16.6 | 10.7 | hypothetical protein GBAA3863 |
| GBAA3866 | 16.6 | 10.7 | iron compound ABC transporter, permease protein |
| GBAA2255 | 15.7 | 9.4 | substrate-binding family protein, putative |
| GBAA3864 | 15.2 | 8.8 | iron compound ABC transporter, ATP-binding protein |
| GBAA0552 | 14.3 | 7.8 | internalin, putative |
| GBAA3533 | 14.0 | 7.5 | iron compound ABC transporter, permease protein |
| GBAA3867 | 14.0 | 7.4 | pseudogene |
| GBAA5240 | 14.0 | 7.4 | L-lactate dehydrogenase |
| GBAA3534 | 13.8 | 7.2 | iron compound ABC transporter, permease protein |
| GBAA3231 | 13.6 | 7.0 | hypothetical protein GBAA3231 |
| GBAA3531 | 13.5 | 6.9 | iron compound ABC transporter, iron compound-binding protein, putative |
| GBAA0766 | 12.7 | 6.2 | nitroreductase family protein |
| GBAA4652 | 12.6 | 6.1 | substrate-binding family protein, putative |
| GBAA4599 | 12.3 | 5.9 | bifunctional acetaldehyde-CoA/alcohol dehydrogenase |
| GBAA3532 | 12.2 | 5.7 | hypothetical protein GBAA3532 |
| GBAA_pXO1_0119 | 11.8 | 5.4 | hypothetical protein (NEAT iron transport domain) |
| GBAA0351 | 11.5 | 5.2 | iron compound ABC transporter, iron compound-binding protein |
| GBAA5239 | 11.5 | 5.2 | hypothetical protein GBAA5239 |
| GBAA2987 | 11.5 | 5.2 | hypothetical protein GBAA2987 |
| GBAA0349 | 11.3 | 5.1 | iron compound ABC transporter, permease protein |
| GBAA0350 | 11.3 | 5.0 | iron compound ABC transporter, permease protein |
| GBAA0352 | 11.1 | 4.9 | pyridine nucleotide-disulfide oxidoreductase family protein |
| GBAA4781 | 10.8 | 4.7 | sodium/hydrogen exchanger family protein |
| GBAA3328 | 10.8 | 4.7 | AraC family transcriptional regulator |
| GBAA_pXO1_0120 | 10.7 | 4.6 | hypothetical protein |
| GBAA0509 | 10.2 | 4.3 | formate acetyltransferase |
| GBAA5410 | 10.2 | 4.3 | pseudogene |
| GBAA5411 | 10.2 | 4.3 | ABC transporter permease/ATP-binding protein |
| GBAA1092 | 10.1 | 4.2 | hypothetical protein GBAA1092 |
| GBAA1943 | 9.1 | 3.7 | cytochrome d ubiquinol oxidase, subunit I |
| GBAA5125 | 9.0 | 3.6 | L-lactate dehydrogenase |
| GBAA1175 | 9.0 | 3.6 | hypothetical protein GBAA1175 |
| GBAA3362 | 8.8 | 3.5 | hypothetical protein GBAA3362 |
| GBAA5631 | 8.5 | 3.4 | pseudogene |
| GBAA1321 | 8.4 | 3.3 | formate/nitrite transporter family protein |
| GBAA4453 | 8.0 | 3.2 | hypothetical protein GBAA4453 |
| GBAA0767 | 8.0 | 3.1 | stage V sporulation protein R |
| GBAA5630 | 7.8 | 3.1 | iron compound ABC transporter, permease protein |
| GBAA3327 | 7.8 | 3.0 | hypothetical protein GBAA3327 |
| GBAA5451 | 7.7 | 3.0 | hypothetical protein GBAA5451 |
| GBAA5298 | 7.7 | 3.0 | NupC family nucleoside transporter |
| GBAA4593 | 7.6 | 3.0 | acetyltransferase |
| GBAA0966 | 7.5 | 2.9 | hypothetical protein GBAA0966 |
| GBAA0618 | 7.5 | 2.9 | iron compound ABC transporter, ATP-binding protein |
| GBAA0616 | 7.5 | 2.9 | iron compound ABC transporter, permease protein |
| GBAA5050 | 7.4 | 2.9 | cytochrome d ubiquinol oxidase, subunit I |
| GBAA0617 | 7.2 | 2.8 | iron compound ABC transporter, permease protein |
| GBAA1757 | 7.1 | 2.8 | hypothetical protein GBAA1757 |
| GBAA5628 | 7.1 | 2.8 | iron compound ABC transporter, iron compound-binding protein |
| GBAA0610 | 6.9 | 2.7 | L-lactate permease |
| GBAA1767 | 6.9 | 2.7 | fumarate hydratase |
| GBAA1987 | 6.9 | 2.7 | small acid-soluble spore protein alpha/beta family protein |
| GBAA5065 | 6.9 | 2.7 | FeoA family protein |
| GBAA5327 | 6.7 | 2.6 | iron compound ABC transporter, ATP-binding protein |
| GBAA5328 | 6.7 | 2.6 | iron compound ABC transporter, permease protein |
| GBAA5064 | 6.7 | 2.6 | ferrous iron transport protein B |
| GBAA4467 | 6.6 | 2.6 | hypothetical protein GBAA4467 |
| GBAA0510 | 6.6 | 2.6 | pyruvate formate-lyase-activating enzyme |
| GBAA5063 | 6.5 | 2.5 | hypothetical protein GBAA5063 |
| GBAA2694 | 6.5 | 2.5 | esterase, putative |
| GBAA5329 | 6.4 | 2.5 | iron compound ABC transporter, permease protein |
| GBAA0167 | 6.3 | 2.5 | hypothetical protein GBAA0167 |
| GBAA2695 | 6.2 | 2.4 | hypothetical protein GBAA2695 |
| GBAA5629 | 6.1 | 2.4 | iron compound ABC transporter, ATP-binding protein |
| GBAA5281 | 6.1 | 2.4 | hypothetical protein GBAA5281 |
| GBAA5330 | 6.1 | 2.4 | iron compound ABC transporter, iron compound-binding protein |
| GBAA2688 | 5.7 | 2.2 | hypothetical protein GBAA2688 |
| GBAA0615 | 5.6 | 2.2 | iron compound ABC transporter, iron compound-binding protein |
| GBAA3730 | 5.6 | 2.2 | holin-like protein |
| GBAA3731 | 5.4 | 2.2 | hypothetical protein GBAA3731 |
| GBAA0416 | 4.7 | 2.0 | eama family protein |
|  |  |  |  |
| 4 hours: |  |  |  |
| **Gene** | **J5** | **FC** | **annotation** |
| GBAA4788 | 23.8 | 160.6 | hypothetical protein GBAA4788 |
| GBAA4789 | 22.4 | 120.7 | cell wall anchor domain-containing protein |
| GBAA4786 | 22.4 | 120.1 | iron compound ABC transporter, iron compound-binding protein |
| GBAA4787 | 22.1 | 112.1 | hypothetical protein GBAA4787 |
| GBAA2368 | 20.5 | 79.7 | 2,3-dihydroxybenzoate-2,3-dehydrogenase |
| GBAA1393 | 20.2 | 74.2 | hypothetical protein GBAA1393 |
| GBAA2369 | 19.6 | 65.4 | isochorismate synthase DhbC |
| GBAA4785 | 19.2 | 60.7 | iron compound ABC transporter, permease protein |
| GBAA4783 | 19.2 | 60.4 | hypothetical protein GBAA4783 |
| GBAA2370 | 18.6 | 53.3 | 2,3-dihydroxybenzoate-AMP ligase |
| GBAA1345 | 18.5 | 52.0 | pseudo - conserved hypothetical protein |
| GBAA1394 | 18.4 | 50.4 | flavodoxin |
| GBAA4784 | 18.2 | 48.5 | iron compound ABC transporter, ATP-binding protein |
| GBAA2374 | 18.1 | 48.2 | EmrB/QacA family drug resistance transporter |
| GBAA2371 | 18.0 | 46.7 | isochorismatase |
| GBAA1395 | 17.8 | 44.9 | hypothetical protein GBAA1395 |
| GBAA2376 | 17.7 | 44.0 | hypothetical protein GBAA2376 |
| GBAA2373 | 17.4 | 41.3 | mbtH-like protein |
| GBAA2372 | 16.7 | 35.5 | nonribosomal peptide synthetase DhbF |
| GBAA2375 | 16.2 | 32.1 | 4'-phosphopantetheinyl transferase, putative |
| GBAA4597 | 16.1 | 30.9 | iron compound ABC transporter, iron compound-binding protein |
| GBAA1346 | 15.9 | 29.9 | internalin, putative |
| GBAA4782 | 14.3 | 21.2 | heme-degrading monooxygenase IsdG |
| GBAA4596 | 13.9 | 19.4 | iron compound ABC transporter, permease protein |
| GBAA1093 | 13.7 | 18.8 | S-layer protein, putative (NEAT - iron transport domain) |
| GBAA4595 | 12.8 | 15.5 | iron compound ABC transporter, ATP-binding protein |
| GBAA0966 | 12.4 | 14.1 | hypothetical protein GBAA0966 |
| GBAA4594 | 12.3 | 13.8 | ankyrin repeat-containing protein |
| GBAA2255 | 12.1 | 13.2 | substrate-binding family protein, putative |
| GBAA0552 | 12.0 | 13.0 | internalin, putative |
| GBAA3595 | 12.0 | 12.9 | BNR repeat-containing protein (up in macrophages) |
| GBAA3596 | 11.0 | 10.6 | flavodoxin |
| GBAA3231 | 10.3 | 9.1 | hypothetical protein GBAA3231 |
| GBAA4781 | 10.0 | 8.5 | sodium/hydrogen exchanger family protein |
| GBAA5690 | 9.5 | 7.6 | murein hydrolase regulator LrgA |
| GBAA_pXO1_0119 | 9.1 | 7.0 | hypothetical protein (NEAT; iron transport domain) |
| GBAA3534 | 8.9 | 6.8 | iron compound ABC transporter, permease protein |
| GBAA5689 | 8.7 | 6.4 | antiholin-like protein LrgB |
| GBAA3533 | 8.7 | 6.4 | iron compound ABC transporter, permease protein |
| GBAA2367 | 8.5 | 6.1 | oxalate:formate antiporter, putative |
| GBAA3531 | 8.5 | 6.1 | iron compound ABC transporter, iron compound-binding protein, putative |
| GBAA_pXO1_0120 | 8.4 | 6.1 | hypothetical protein |
| GBAA1092 | 8.2 | 5.7 | hypothetical protein GBAA1092 |
| GBAA0766 | 8.1 | 5.7 | nitroreductase family protein |
| GBAA3866 | 7.8 | 5.3 | iron compound ABC transporter, permease protein |
| GBAA3532 | 7.6 | 5.1 | hypothetical protein GBAA3532 |
| GBAA3867 | 7.5 | 4.9 | pseudogene |
| GBAA3865 | 7.4 | 4.9 | iron compound ABC transporter, permease protein |
| GBAA2688 | 7.3 | 4.8 | hypothetical protein GBAA2688 |
| GBAA3863 | 7.3 | 4.8 | hypothetical protein GBAA3863 |
| GBAA4652 | 7.0 | 4.5 | substrate-binding family protein, putative |
| GBAA3864 | 6.7 | 4.2 | iron compound ABC transporter, ATP-binding protein (bacilobactin transport?) |
| GBAA3328 | 6.6 | 4.1 | AraC family transcriptional regulator |
| GBAA4453 | 6.5 | 4.0 | hypothetical protein GBAA4453 |
| GBAA2987 | 6.4 | 3.9 | hypothetical protein GBAA2987 |
| GBAA5411 | 6.2 | 3.8 | ABC transporter permease/ATP-binding protein |
| GBAA5298 | 5.9 | 3.5 | NupC family nucleoside transporter |
| GBAA0351 | 5.7 | 3.4 | iron compound ABC transporter, iron compound-binding protein |
| GBAA0792 | 5.7 | 3.4 | PTS system, cellobiose-specific IIB component |
| GBAA3327 | 5.6 | 3.3 | hypothetical protein GBAA3327 |
| GBAA1757 | 5.5 | 3.2 | hypothetical protein GBAA1757 |
| GBAA0615 | 5.4 | 3.2 | iron compound ABC transporter, iron compound-binding protein |
| GBAA4593 | 5.2 | 3.0 | acetyltransferase |
| GBAA3362 | 5.1 | 3.0 | hypothetical protein GBAA3362 |
| GBAA5240 | 5.1 | 3.0 | L-lactate dehydrogenase |
| GBAA5410 | 5.0 | 2.9 | pseudogene |
| GBAA1943 | 5.0 | 2.9 | cytochrome d ubiquinol oxidase, subunit I |
| GBAA0349 | 4.9 | 2.8 | iron compound ABC transporter, permease protein |
| GBAA0350 | 4.9 | 2.8 | iron compound ABC transporter, permease protein |
| GBAA0352 | 4.8 | 2.8 | pyridine nucleotide-disulfide oxidoreductase family protein |
| GBAA2956 | 4.7 | 2.7 | chorismate synthase |
| GBAA5631 | 4.6 | 2.7 | pseudogene |
| GBAA5330 | 4.6 | 2.7 | iron compound ABC transporter, iron compound-binding protein |
| GBAA1175 | 4.6 | 2.7 | hypothetical protein GBAA1175 |
| GBAA_pXO1_0121 | 4.6 | 2.7 | hypothetical protein |
| GBAA5647 | 4.5 | 2.6 | hypothetical protein GBAA5647 |
| GBAA0616 | 4.5 | 2.6 | iron compound ABC transporter, permease protein |
| GBAA1086 | 4.4 | 2.6 | LacI family sugar-binding transcriptional regulator |
| GBAA2366 | 4.4 | 2.6 | hypothetical protein GBAA2366 |
| GBAA2851 | 4.4 | 2.6 | pseudogene |
| GBAA2694 | 4.4 | 2.6 | esterase, putative |
| GBAA0239 | 4.4 | 2.5 | hypothetical protein GBAA0239 |
| GBAA3873 | 4.4 | 2.5 | hypothetical protein GBAA3873 |
| GBAA0791 | 4.3 | 2.5 | PTS system, cellobiose-specific IIA component |
| GBAA5072 | 4.3 | 2.5 | hypothetical protein GBAA5072 |
| GBAA2955 | 4.3 | 2.5 | histidinol-phosphate aminotransferase |
| GBAA5328 | 4.2 | 2.5 | iron compound ABC transporter, permease protein |
| GBAA5329 | 4.2 | 2.5 | iron compound ABC transporter, permease protein |
| GBAA5239 | 4.1 | 2.4 | hypothetical protein GBAA5239 |
| GBAA1767 | 4.1 | 2.4 | fumarate hydratase |
| GBAA5125 | 4.0 | 2.4 | L-lactate dehydrogenase |
| GBAA0793 | 4.0 | 2.4 | PTS system, cellobiose-specific IIC component |
| GBAA0238 | 4.0 | 2.3 | hypothetical protein GBAA0238 |
| GBAA2695 | 3.9 | 2.3 | hypothetical protein GBAA2695 |
| GBAA5065 | 3.9 | 2.3 | FeoA family protein |
| GBAA5528 | 3.8 | 2.3 | stage II sporulation protein D |
| GBAA0610 | 3.8 | 2.3 | L-lactate permease |
| GBAA4905 | 3.8 | 2.3 | hypothetical protein GBAA4905 |
| GBAA5281 | 3.8 | 2.3 | hypothetical protein GBAA5281 |
| GBAA0767 | 3.8 | 2.2 | stage V sporulation protein R |
| GBAA5327 | 3.8 | 2.2 | iron compound ABC transporter, ATP-binding protein |
| GBAA3554 | 3.8 | 2.2 | penicillin-binding protein, putative |
| GBAA0617 | 3.7 | 2.2 | iron compound ABC transporter, permease protein |
| GBAA1458 | 3.7 | 2.2 | hypothetical protein GBAA1458 |
| GBAA_pXO1_0122 | 3.7 | 2.2 | pseudogene |
| GBAA5630 | 3.7 | 2.2 | iron compound ABC transporter, permease protein |
| GBAA0618 | 3.6 | 2.2 | iron compound ABC transporter, ATP-binding protein |
| GBAA2744 | 3.6 | 2.2 | acetyltransferase |
| GBAA2954 | 3.6 | 2.1 | prephenate dehydrogenase |
| GBAA4592 | 3.5 | 2.1 | acetyltransferase |
| GBAA0312 | 3.5 | 2.1 | ABC transporter ATP-binding protein |
| GBAA2425 | 3.5 | 2.1 | hypothetical protein GBAA2425 |
| GBAA2743 | 3.5 | 2.1 | hypothetical protein GBAA2743 |
| GBAA0794 | 3.4 | 2.1 | hypothetical protein GBAA0794 |
| GBAA3535 | 3.4 | 2.1 | hypothetical protein GBAA3535 |
| GBAA1988 | 3.4 | 2.1 | pseudo - multidrug resistance protein, putative |
| GBAA1110 | 3.4 | 2.1 | pseudo - Ser/Thr protein phosphatase family protein |
| GBAA2016 | 3.3 | 2.0 | luciferase family protein |
| GBAA3438 | 3.3 | 2.0 | alcohol dehydrogenase, zinc-containing |
| GBAA3663 | 3.3 | 2.0 | anaerobic ribonucleoside triphosphate reductase |
| GBAA4766 | 3.3 | 2.0 | iron compound ABC transporter, iron compound-binding protein |
| GBAA4259 | 3.3 | 2.0 | hypothetical protein GBAA4259 |
| GBAA4467 | 3.2 | 2.0 | hypothetical protein GBAA4467 |
| GBAA0346 | 3.2 | 2.0 | 5-methylribose kinase |
| GBAA0167 | 3.2 | 2.0 | hypothetical protein GBAA0167 |
| GBAA2958 | 3.2 | 2.0 | bifunctional 3-deoxy-7-phosphoheptulonate synthase/chorismate mutase |
| GBAA_pXO1_0149 | 3.2 | 2.0 | hypothetical protein |
| GBAA2286 | 3.2 | 2.0 | hypothetical protein GBAA2286 |
| GBAA2633 | 3.2 | 2.0 | hypothetical protein GBAA2633 |
| GBAA1789 | 3.2 | 2.0 | RNA polymerase sigma factor SigW |
| GBAA0416 | 3.2 | 2.0 | eama family protein |
| GBAA5386 | 3.2 | 2.0 | hypothetical protein GBAA5386 |
| GBAA0544 | 3.1 | 2.0 | hypothetical protein GBAA0544 |
| GBAA2693 | 3.1 | 2.0 | hypothetical protein GBAA2693 |
| GBAA3576 | 3.1 | 2.0 | hypothetical protein GBAA3576 |
| GBAA5064 | 3.1 | 2.0 | ferrous iron transport protein B |
