# Supplementary material for: Transcriptional Profiling of Bacillus anthracis Sterne (34F2) during Iron Starvation
Source: PLoS One. 2009 Sep 21;4(9):e6988. doi: 10.1371/journal.pone.0006988 (PMC2742718; doi:10.1371/journal.pone.0006988)
Supplement: Table S2 — Genes down-regulated during iron starvation. mRNA transcripts downregulated in IDM as compared to regulation in IRM (0.24 MB DOC) [file pone.0006988.s002.doc]

| **Table S2: Genes down-regulated during iron starvation.** | | | |
| --- | --- | --- | --- |
|  |  |  |  |
| 2 hours: |  |  |  |
| **Gene** | **J5** | **FC** | **annotation** |
| GBAA0235 | -17.1 | -2.3 | oligopeptide ABC transporter, ATP-binding protein |
| GBAA0234 | -16.2 | -2.2 | oligopeptide ABC transporter, ATP-binding protein |
| GBAA0776 | -15.5 | -2.1 | rhodanese domain-containing protein |
| GBAA0410 | -15.3 | -2.1 | heavy metal-transporting ATPase |
| GBAA0779 | -15.1 | -2.1 | rhodanese-like domain-containing protein |
| GBAA0777 | -14.7 | -2.0 | metallo-beta-lactamase family protein |
|  |  |  |  |
| 3 hours: |  |  |  |
| **Gene** | **J5** | **FC** | **annotation** |
| GBAA0410 | -10.8 | -4.7 | heavy metal-transporting ATPase |
| GBAA0204 | -10.5 | -4.5 | pseudogene |
| GBAA0202 | -9.6 | -3.9 | molybdenum ABC transporter permease |
| GBAA4028 | -7.3 | -2.9 | aspartate carbamoyltransferase catalytic subunit |
| GBAA4029 | -7.2 | -2.8 | uracil permease |
| GBAA3029 | -7.0 | -2.7 | succinylornithine transaminase, putative |
| GBAA0203 | -6.9 | -2.7 | hypothetical protein GBAA0203 |
| GBAA5705 | -6.4 | -2.5 | guanosine 5'-monophosphate oxidoreductase |
| GBAA2301 | -6.3 | -2.5 | hypothetical protein GBAA2301 |
| GBAA4027 | -6.0 | -2.4 | dihydroorotase |
| GBAA2300 | -5.9 | -2.3 | L-lysine 2,3-aminomutase |
| GBAA1159 | -5.7 | -2.3 | catalase |
| GBAA4330 | -5.6 | -2.2 | hypothetical protein GBAA4330 |
| GBAA5300 | -5.2 | -2.1 | sodium/alanine symporter family protein |
| GBAA0423 | -5.2 | -2.1 | fumarate hydratase, class I |
| GBAA4780 | -5.1 | -2.1 | hypothetical protein GBAA4780 |
| GBAA4026 | -5.1 | -2.1 | carbamoyl phosphate synthase small subunit |
| GBAA5693 | -5.0 | -2.1 | major facilitator family transporter |
| GBAA0563 | -5.0 | -2.0 | putative lipoprotein |
| GBAA4574 | -4.9 | -2.0 | alkaline phosphatase |
| GBAA1503 | -4.9 | -2.0 | ferredoxin |
| GBAA4030 | -4.7 | -2.0 | pyrimidine regulatory protein PyrR |
| GBAA4573 | -4.7 | -2.0 | putative lipoprotein |
|  |  |  |  |
|  |  |  |  |
| 4 hours: |  |  |  |
| **Gene** | **J5** | **FC** | **annotation** |
| GBAA2145 | -10.0 | -8.5 | nitrite reductase |
| GBAA3497 | -9.2 | -7.1 | hydroxylamine reductase |
| GBAA4028 | -8.1 | -5.6 | aspartate carbamoyltransferase catalytic subunit |
| GBAA2144 | -8.0 | -5.5 | bifunctional uroporphyrinogen-III methyltransferase/uroporphyrinogen-III synthase |
| GBAA2133 | -7.8 | -5.3 | molybdenum cofactor biosynthesis protein A |
| GBAA4027 | -7.6 | -5.1 | dihydroorotase |
| GBAA2134 | -7.6 | -5.0 | thiamine/molybdopterin biosynthesis MoeB-like protein |
| GBAA2301 | -7.6 | -5.0 | hypothetical protein GBAA2301 |
| GBAA2146 | -7.5 | -5.0 | nitrite reductase |
| GBAA4026 | -7.4 | -4.9 | carbamoyl phosphate synthase small subunit |
| GBAA2300 | -7.3 | -4.7 | L-lysine 2,3-aminomutase |
| GBAA2137 | -7.1 | -4.6 | molybdopterin converting factor, subunit 1 |
| GBAA0203 | -7.1 | -4.5 | hypothetical protein GBAA0203 |
| GBAA2125 | -7.0 | -4.5 | respiratory nitrate reductase, alpha subunit |
| GBAA2136 | -6.8 | -4.3 | molybdopterin converting factor, subunit 2 |
| GBAA0202 | -6.7 | -4.2 | molybdenum ABC transporter permease |
| GBAA2135 | -6.6 | -4.1 | molybdopterin biosynthesis protein moea |
| GBAA5481 | -6.6 | -4.1 | hypothetical protein GBAA5481 |
| GBAA0410 | -6.6 | -4.1 | heavy metal-transporting ATPase |
| GBAA3029 | -6.5 | -4.0 | succinylornithine transaminase, putative |
| GBAA4029 | -6.4 | -3.9 | uracil permease |
| GBAA3496 | -6.4 | -3.9 | hypothetical protein GBAA3496 |
| GBAA4025 | -6.3 | -3.8 | carbamoyl phosphate synthase large subunit |
| GBAA4024 | -6.2 | -3.7 | dihydroorotate dehydrogenase electron transfer subunit |
| GBAA0204 | -6.1 | -3.7 | pseudo - modA - molybdenum ABC transporter, molybdenum-binding protein |
| GBAA0716 | -6.0 | -3.6 | phosphate ABC transporter permease |
| GBAA4021 | -6.0 | -3.6 | orotate phosphoribosyltransferase |
| GBAA0655 | -5.8 | -3.5 | hypothetical protein GBAA0655 |
| GBAA4023 | -5.8 | -3.5 | dihydroorotate dehydrogenase 1B |
| GBAA2381 | -5.8 | -3.4 | sodium/alanine symporter family protein |
| GBAA4573 | -5.7 | -3.4 | putative lipoprotein |
| GBAA2143 | -5.7 | -3.4 | CbiX domain-containing protein |
| GBAA0715 | -5.5 | -3.3 | phosphate ABC transporter phosphate-binding protein |
| GBAA0717 | -5.4 | -3.2 | phosphate ABC transporter permease |
| GBAA1451 | -5.3 | -3.1 | hypothetical protein GBAA1451 |
| GBAA4022 | -5.3 | -3.1 | orotidine 5'-phosphate decarboxylase |
| GBAA5300 | -5.2 | -3.0 | sodium/alanine symporter family protein |
| GBAA3646 | -5.2 | -3.0 | pseudogene |
| GBAA0689 | -5.1 | -3.0 | proton/peptide symporter family protein |
| GBAA3845 | -4.8 | -2.8 | hypothetical protein GBAA3845 |
| GBAA4574 | -4.8 | -2.8 | alkaline phosphatase |
| GBAA1099 | -4.7 | -2.8 | hypothetical protein GBAA1099 |
| GBAA4492 | -4.7 | -2.7 | phosphate transporter PhoU |
| GBAA2914 | -4.7 | -2.6 | hypothetical protein GBAA2914 |
| GBAA2609 | -4.7 | -2.7 | GntR family transcriptional regulator |
| GBAA2915 | -4.7 | -2.7 | hypothetical protein GBAA2915 |
| GBAA4030 | -4.6 | -2.7 | pyrimidine regulatory protein PyrR |
| GBAA0910 | -4.6 | -2.7 | oligopeptide ABC transporter, permease protein |
| GBAA2913 | -4.6 | -2.7 | hypothetical protein GBAA2913 |
| GBAA2913 | -4.6 | -2.7 | pseudogene |
| GBAA5286 | -4.6 | -2.7 | sodium/hydrogen exchanger family protein |
| GBAA2126 | -4.6 | -2.7 | respiratory nitrate reductase, beta subunit |
| GBAA0909 | -4.5 | -2.6 | oligopeptide ABC transporter, permease protein |
| GBAA2914 | -4.5 | -2.7 | pseudogene |
| GBAA4826 | -4.5 | -2.6 | hypothetical protein GBAA4826 |
| GBAA4773 | -4.5 | -2.6 | hypothetical protein GBAA4773 |
| GBAA0845 | -4.5 | -2.6 | amino acid permease family protein |
| GBAA1098 | -4.5 | -2.6 | wall-associated domain-containing protein |
| GBAA5686 | -4.4 | -2.6 | AcrB/AcrD/AcrF family transporter |
| GBAA1097 | -4.4 | -2.6 | hypothetical protein GBAA1097 |
| GBAA5261 | -4.4 | -2.5 | amino acid permease family protein |
| GBAA4771 | -4.4 | -2.5 | hypothetical protein GBAA4771 |
| GBAA3833 | -4.3 | -2.5 | glutamine synthetase, type I |
| GBAA5288 | -4.3 | -2.5 | hypothetical protein GBAA5288 |
| GBAA5287 | -4.3 | -2.5 | TrkA domain-containing protein |
| GBAA5693 | -4.2 | -2.5 | major facilitator family transporter |
| GBAA2142 | -4.2 | -2.4 | precorrin-2 dehydrogenase |
| GBAA2911 | -4.2 | -2.4 | hypothetical protein GBAA2911 |
| GBAA1337 | -4.1 | -2.4 | iron compound ABC transporter, iron compound-binding protein, putative |
| GBAA2127 | -4.1 | -2.4 | nitrate reductase delta chain |
| GBAA4770 | -4.1 | -2.4 | hypothetical protein GBAA4770 |
| GBAA0912 | -4.0 | -2.4 | oligopeptide ABC transporter, ATP-binding protein |
| GBAA1423 | -4.0 | -2.4 | isopropylmalate isomerase small subunit |
| GBAA0212 | -4.0 | -2.3 | hypothetical protein GBAA0212 |
| GBAA1450 | -4.0 | -2.3 | proton/glutamate symporter family protein |
| GBAA0796 | -4.0 | -2.3 | hypothetical protein GBAA0796 |
| GBAA4608 | -3.9 | -2.3 | uridine kinase |
| GBAA0797 | -3.9 | -2.3 | ABC transporter permease |
| GBAA1811 | -3.9 | -2.3 | aspartate kinase |
| GBAA4667 | -3.9 | -2.3 | ABC transporter permease |
| GBAA0501 | -3.9 | -2.3 | PTS system, N-acetylglucosamine-specific IIBC component, putative |
| GBAA2912 | -3.9 | -2.3 | hypothetical protein GBAA2912 |
| GBAA0911 | -3.9 | -2.3 | pseudo - oligopeptide ABC transporter, ATP-binding protein |
| GBAA4774 | -3.8 | -2.3 | bacitracin ABC transporter, ATP-binding protein |
| GBAA5301 | -3.8 | -2.3 | sodium/alanine symporter family protein |
| GBAA5162 | -3.8 | -2.3 | hypothetical protein GBAA5162 |
| GBAA1735 | -3.8 | -2.3 | ABC transporter substrate-binding protein |
| GBAA0206 | -3.8 | -2.2 | hypothetical protein GBAA0206 |
| GBAA0939 | -3.8 | -2.2 | hypothetical protein GBAA0939 |
| GBAA0587 | -3.8 | -2.2 | acetyltransferase |
| GBAA5583 | -3.7 | -2.2 | CTP synthetase |
| GBAA1159 | -3.7 | -2.2 | catalase |
| GBAA1087 | -3.7 | -2.2 | hypothetical protein GBAA1087 |
| GBAA1422 | -3.7 | -2.2 | isopropylmalate isomerase large subunit |
| GBAA3076 | -3.7 | -2.2 | lysine-specific permease |
| GBAA1751 | -3.7 | -2.2 | asparagine synthetase, glutamine-hydrolyzing |
| GBAA1412 | -3.7 | -2.2 | hypothetical protein GBAA1412 |
| GBAA1408 | -3.7 | -2.2 | proton/glutamate symporter family protein |
| GBAA2128 | -3.7 | -2.2 | respiratory nitrate reductase, gamma subunit |
| GBAA1117 | -3.6 | -2.2 | hypothetical protein GBAA1117 |
| GBAA0151 | -3.6 | -2.2 | hypothetical protein GBAA0151 |
| GBAA0798 | -3.6 | -2.2 | ABC transporter ATP-binding protein |
| GBAA1100 | -3.6 | -2.2 | hypothetical protein GBAA1100 |
| GBAA1605 | -3.6 | -2.2 | cation transporter, putative |
| GBAA4772 | -3.6 | -2.1 | hypothetical protein GBAA4772 |
| GBAA4610 | -3.5 | -2.1 | U32 family peptidase |
| GBAA0232 | -3.5 | -2.1 | oligopeptide ABC transporter, permease protein |
| GBAA1295 | -3.5 | -2.1 | immune inhibitor a metalloprotease |
| GBAA4780 | -3.5 | -2.1 | hypothetical protein GBAA4780 |
| GBAA1734 | -3.5 | -2.1 | ABC transporter ATP-binding protein |
| GBAA0194 | -3.5 | -2.1 | oligopeptide ABC transporter, oligopeptide-binding protein, putative |
| GBAA0789 | -3.5 | -2.1 | hypothetical protein GBAA0789 |
| GBAA0142 | -3.4 | -2.1 | pseudo - tRNA pseudouridine synthase A |
| GBAA1338 | -3.4 | -2.1 | pseudo - ABC transporter, ATP-binding protein |
| GBAA1564 | -3.4 | -2.1 | aspartate alpha-decarboxylase |
| GBAA5589 | -3.4 | -2.1 | acetyl-CoA acetyltransferase |
| GBAA4609 | -3.4 | -2.1 | U32 family peptidase |
| GBAA2390 | -3.4 | -2.1 | ABC transporter permease |
| GBAA2637 | -3.4 | -2.1 | penicillin-binding protein |
| GBAA1421 | -3.3 | -2.0 | 3-isopropylmalate dehydrogenase |
| GBAA2610 | -3.3 | -2.0 | D-alanine--D-alanine ligase |
| GBAA1160 | -3.3 | -2.0 | pseudo - ammonium transporter |
| GBAA3986 | -3.3 | -2.0 | chromosome segregation SMC protein |
| GBAA1606 | -3.3 | -2.0 | 5'-3' exonuclease family protein |
| GBAA3854 | -3.3 | -2.0 | extracellular exochitinase Chi36 |
| GBAA4535 | -3.3 | -2.0 | hypothetical protein GBAA4535 |
| GBAA5588 | -3.3 | -2.0 | 3-hydroxybutyryl-CoA dehydrogenase |
| GBAA0950 | -3.3 | -2.0 | hypothetical protein GBAA0950 |
| GBAA0554 | -3.3 | -2.0 | glycine betaine transporter |
| GBAA2138 | -3.3 | -2.0 | nitrate transporter |
| GBAA1653 | -3.3 | -2.0 | glyoxalase family protein |
| GBAA5590 | -3.3 | -2.0 | ferredoxin, 4Fe-4S |
| GBAA1736 | -3.3 | -2.0 | ABC transporter permease |
| GBAA3020 | -3.3 | -2.0 | major facilitator family transporter |
| GBAA1420 | -3.3 | -2.0 | 2-isopropylmalate synthase |
| GBAA0257 | -3.3 | -2.0 | hypothetical protein GBAA0257 |
| GBAA0233 | -3.2 | -2.0 | oligopeptide ABC transporter, permease protein |
| GBAA4707 | -3.2 | -2.0 | hypothetical protein GBAA4707 |
| GBAA0908 | -3.2 | -2.0 | oligopeptide ABC transporter, oligopeptide-binding protein |
| GBAA4993 | -3.2 | -2.0 | sodium/hydrogen exchanger family protein |
| GBAA0799 | -3.2 | -2.0 | hypothetical protein GBAA0799 |
| GBAA0835 | -3.2 | -2.0 | multidrug resistance protein |
| GBAA0200 | -3.1 | -2.0 | transporter, putative |
| GBAA1812 | -3.1 | -2.0 | hypothetical protein GBAA1812 |
| GBAA0207 | -3.1 | -2.0 | hypothetical protein GBAA0207 |
